# Systematic review and meta-analysis of the effectiveness of continuous glucose monitoring (CGM) on glucose control in diabetes

**DOI:** 10.1186/1758-5996-5-39

**Published:** 2013-07-23

**Authors:** Nalinee Poolsup, Naeti Suksomboon, Aye Mon Kyaw

**Affiliations:** 1 Department of Pharmacy, Faculty of Pharmacy, Mahidol University, Bangkok, Thailand; 2 Department of Pharmacy, Faculty of Pharmacy, Silpakorn University, Nakhon-Pathom, Thailand

**Keywords:** Systematic review, Continuous glucose monitoring (CGM), T1DM, Type 1 diabetes, T2DM, Type 2 diabetes, SMBG, Self monitoring of blood glucose

## Abstract

Diabetes mellitus is a chronic disease that necessitates continuing treatment and patient self-care education. Monitoring of blood glucose to near normal level without hypoglycemia becomes a challenge in the management of diabetes. Although self monitoring of blood glucose (SMBG) can provide daily monitoring of blood glucose level and help to adjust therapy, it cannot detect hypoglycemic unawareness and nocturnal hypoglycemia which occurred mostly in T1DM pediatrics. Continuous glucose monitoring (CGM) offers continuous glucose data every 5 minutes to adjust insulin therapy especially for T1DM patients and to monitor lifestyle intervention especially for T2DM patients by care providers or even patients themselves. The main objective of this study was to assess the effects of continuous glucose monitoring (CGM) on glycemic control in Type 1 diabetic pediatrics and Type 2 diabetic adults by collecting randomized controlled trials from MEDLINE (pubmed), SCOPUS, CINAHL, Web of Science and The Cochrane Library up to May 2013 and historical search through the reference lists of relevant articles. There are two types of CGM device: real-time CGM and retrospective CGM and both types of the device were included in the analysis. In T1DM pediatrics, CGM use was no more effective than SMBG in reducing HbA1c [mean difference – 0.13% (95% CI -0.38% to 0.11%,]. This effect was independent of HbA1c level at baseline. Subgroup analysis indicated that retrospective CGM was not superior to SMBG [mean difference -0.05% (95% CI -0.46% to 0.35%)]. In contrast, real-time CGM revealed better effect in lowering HbA1c level compared with SMBG [mean difference -0.18% (95% CI -0.35% to -0.02%, p = 0.02)]. In T2DM adults, significant reduction in HbA1c level was detected with CGM compared with SMBG [mean difference – 0.31% (95% CI -0.6% to -0.02%, p = 0.04)].

This systematic review and meta-analysis suggested that real-time CGM can be more effective than SMBG in T1DM pediatrics, though retrospective CGM was not. CGM provided better glycemic control in T2DM adults compared with SMBG.

## Introduction

The global prevalence of diabetes by International Diabetes Federation (IDF) estimation shows that there are 366 million people with diabetes in 2011, and this is expected to rise to 552 million by 2030 [1]. Diabetes caused 4.6 million deaths in 2011 [2]. Most of people afflicted with diabetes (85 – 95%) have type 2 diabetes mellitus [3]. Forty-four percent of people with type 2 diabetes show hemoglobin A1C higher than the generally accepted target [4]. Recent studies have established that improved glycemic control by means of intensive diabetes management decreases the risk of long-term microvascular complications in both type 1 and type 2 diabetic patients [5]. However, the major problem with an intensive diabetes treatment is the increased risk of hypoglycemic events. Subjects in the intensive management group of the Diabetes Control and Complications Trial (DCCT) experienced severe hypoglycemia two to three times more often than subjects receiving conventional therapy [6]. The fear of hypoglycemia often leads patients to forget the fatal consequences of long-term complications resulting in loss of control and cognitive dysfunction [7]. Therefore, in addition to intensive treatment, self management of blood glucose to normal or within normal limits is a crucial aspect.

Self monitoring of blood glucose (SMBG) has been shown to be as effective in insulin-treated type 1 and type 2 diabetes. Although the effect of SMBG already demonstrated in some meta-analysis [8,9], it is not recommended as regularly use in non-insulin treated type 2 diabetes. SMBG fails to detect nocturnal hypoglycemia and asymptomatic hypoglycemia even in patients with good control of HbA1c values and it needs multiple blood samples throughout the day. In addition, SMBG gives a single instant reading without any information on glucose trends and thus may miss important and significant glucose fluctuations [10-12]. Continuous glucose monitoring (CGM) measures interstitial fluid every 10 seconds and an average glucose value is recorded every five minutes 24 hours a day. This gives a more accurate pattern of daily glucose fluctuations allowing identification of the glycemic effect of food, physical activity, insulin and different medication types and doses aiding in better self management with avoiding unrecognized hypoglycemia [13].

Although some randomized trials show the benefit of CGM use over SMBG in type 1 diabetic pediatric population [14,15], previous meta-analysis on type 1 diabetic pediatric patients failed to show the effectiveness of CGM on the decline of HbA1c level compared with SMBG [16], further evidence is still needed to provide effectiveness of CGM on pediatric type 1 diabetic population. Only one meta-analysis reported that the effectiveness of CGM on type 2 diabetic patients was superior to SMBG use [17]. However, the strength of evidence of reducing HbA1c in that population is limited because the number of patients in the included studies was rather low. In our meta-analysis, the effectiveness of CGM use over SMBG was evaluated in Type 1 diabetic pediatrics and Type 2 diabetic adults to provide a comprehensive and quantitative synthesis of evidence from all randomized controlled trials (RCTs) and to make clear the conflict about CGM effectiveness in these populations.

## Methods

### Data Sources

Reports of randomized controlled trials of CGM aimed for good glycemic control in type 1 diabetic and type 2 diabetic patients are identified through a systematic literature search of MEDLINE (Pubmed), Scopus, CINAHL, Web of Science and The Cochrane Library. The bibliographic databases were searched from the inception to May 2013. The following MeSH terms were used; diabetes mellitus and continuous glucose monitoring. This was followed by keyword search using as keywords continuous monitoring of blood glucose, CGM, insulin-dependent diabetes mellitus, IDDM, non-insulin dependent diabetes mellitus and NIDDM. Historical search of reference lists of relevant randomized controlled trials, systematic and narrative reviews was also undertaken. No language restriction was imposed.

### Inclusion criteria and exclusion criteria

To be included in the systematic review, studies had to be

a) randomized controlled trials comparing currently available CGM device with SMBG in patients with type 1 diabetes pediatrics (≤ 18 yrs) or type 2 diabetes adults (≥ 18 yrs),

b) of at least 8 weeks duration, and

c) reporting HbA1c as an outcome measure.

We excluded studies involving

a) pregnant women,

b) critically ill patients, post-surgery, post-transplant and ICU patients.

### Data extraction and study quality assessment

Data from individual studies were abstracted and study quality was evaluated independently by two authors using a standardized form. The disagreements were resolved by a third author. The methodological quality of each study was assessed using Maastricht Amsterdam scale [18], which has been developed based on the scale of Jadad et al [19] and the Delphi list [20]. These 12 items evaluated the internal validity of the study results. Each item had a rating scale of “yes,” “no,” or “unsure.” If bias was unlikely, the item was rated positive. If bias was likely, the item was rated as negative. Then, if information concerning the item was not available, it was rated with “unsure.” Out of 12 items, only 11 items were used to assess the quality of studies because one item that investigates patient compliance is not applicable to CGM intervention. Studies that met at least 6 of 11 quality criteria were of high quality. Those scoring less than 6 of the criteria were of low quality or having high risk of bias.

### Statistical analysis

Outcome of interest was HbA1c. Treatment effect was estimated with mean difference in the final values of HbA1c between the CGM group and the SMBG group. In a randomized trial, a comparison of final measurement can usually be assumed to provide the same estimate as the comparison of changes from baseline. Random effects model was used to combine the results of individual studies when Q-statistic test was significant at the level of 0.1, otherwise the fixed effects model was used. Heterogeneity was also determined by I^2^. Substantial heterogeneity was represented by I^2^ of 50% or more [21]. Subgroup analysis was carried out based on the data presentation of CGM device [real-time (RT) CGM and retrospective (r) CGM] and study quality. Moreover, subgroup analysis based on baseline HbA1c level (< 8%, 8-10%, and >10%) was also performed to assess the impact of baseline HbA1c level on the effectiveness of CGM. Sensitivity analysis was conducted by excluding studies reporting lower number of usable CGM data. The statistical analysis was undertaken with Review Manager (Revman®) program version 5.2.3 (Cochrance collaboration, Oxford UK). A funnel plot and Egger regression test [22] were used to assess publication bias.

## Results

### Study characteristics

**Figure 1:**
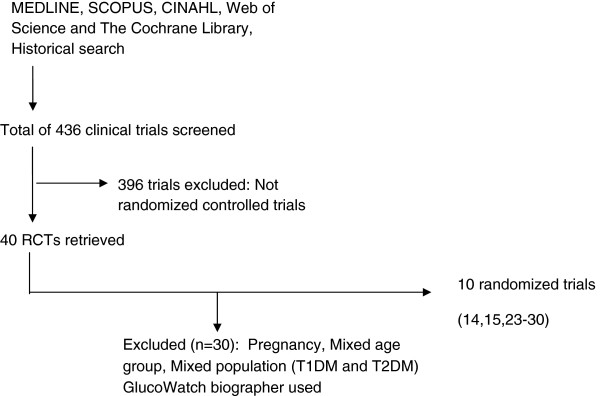
Summary of trial flow (Type 1 diabetic pediatrics).

In pediatric Type 1 diabetes, ten RCTs involving 817 participants met our predefined inclusion criteria. The diagram of data extraction is illustrated in Figure 1. The characteristics of included trials are summarized in Table 1. Seven studies are regarded as high quality [14,15,23-27] and three studies are regarded as low quality [28-30]. Retrospective CGM (r-CGM) was used in 5 studies [23-25,28,29] and Real-Time CGM (RT-CGM) was used in 5 studies [14,15,26,27,30]. Duration of study of all trials ranged from 3 months to 12 months. Five studies [24-26,28,30] included participants with insulin pump therapy or insulin injection therapy, three studies [14,23,29] included only participants with insulin injection therapy and two studies [15,27] included only participants with insulin pump therapy. Among ten studies, four studies were multi-centered [14,15,26,27]. There were two single-blinded studies [24,30] and one double-blinded study [23]. Two out of ten studies were cross-over randomized trials [15,23].

**Table 1:** Characteristics of included studies in Type 1 diabetic pediatrics

**Study**	**Country**	**Duration**	**N**	**Intervention**	**CGM Use**	**SMBG Use**	**Main Outcomes**
Chase [28]	USA	3 months	11	The MiniMed CGM vs. SMBG	18 total sensor days within 30- day (total 6 times) + 4 times SMBG tests	Minimum of 4 daily SMBG tests	• HbA1c
			I:5				• Number of hypoglycemic events
			C:6				• Number of insulin dosage changes
							• Fear of hypoglycemia
Deiss [23]	Germany	3 months	30	The MiniMed CGM vs. SMBG	3 days of CGM every 6 weeks over 12 weeks. (total 2 times) (once open, once blinded) + at least 5 times SMBG tests	At least 5 times per day	• HbA1c
			I:15				
			C:15				
Lagarde [24]	USA	6 months	27	RT-CGM vs. SMBG	3 days of CGM (Open) every 2 months. (total 3 times) +Usual practice of monitoring BG	3 days of CGM (Blinded) at 0,2, and 4 months +	• HbA1c
			I:18				• AUC for glucose < 70 mg/dL
			C:9			Usual practice of monitoring BG	• Duration and severity of hypoglycemia
					before meals, at bedtime and at 2:00 hours once weekly.	before meals, at bedtime and at 2:00 hours once weekly.	
Yates [25]	Australia	3 months	36	The MiniMed CGMvs.SMBG	3 days of CGM every 3 weeks over 3 months (total 4 times) + at least 4 times SMBG	4 to 6 times daily	• HbA1c
			I: 19				• Fructosamine
			C:17				
JDRF [26]	England	26 weeks	114	CGMS	Use the device on a daily basis + 4times SMBG	At least 4 times daily	• HbA1c
			I:56	(DexCom or Medtronic or FreeStyle Navigator) vs. SMBG			• The amount of time in hypoglycemic and hyperglycemic per day
			C:58				• relative reduction of 10% or more in the mean glycated hemoglobin level
Bergenstal [14]	England	12 months	156	CGM (Minimed paradigm) Vs. SMBG (Sensor augmented pump vs. MDI)	1-week period/ 6 months + SMBG	Sensor glucose values are collected for 1 week periods at Baseline, 6 months and 1 years (Not Display data)	• HbA1c
			I: 78				• Severe rates of hypoglycemia
			C: 78				
Kondonouri [27]	France	12 months	154	CGM (MiniMed paradigm) vs. SMBG	Use on a daily basis, replace the sensors every 3 days + SMBG (at least 4 times/day)	At least 4 times daily	• HbA1c
			I: 76				
			C: 78				
Battelino [15]	Slovenia	6 month	72	CGM (Guardian REAL-Time) vs. SMBG	Used on a daily basis + SMBG	SMBG	• HbA1c
			I/C:37				• Changes in glycaemic patterns
			C/I:35				• Changes in the time spent in hypoglycemia, hyperglycemia, and euglycaemia
Bukara-Radujkovic [29]	BosniaHerzegovina	6 months	80	CGM (Medtronic Minimed) vs. SMBG	3 days of CGM (only one time) + at least 4 daily SMBG (before and after each main meal, at bedtime and during the night at 2 a.m and 5 a.m)	At least 4 daily SMBG (before and after each main meal, at bedtime and during the night at 2 a.m and 5 a.m)	• HbA1c
			I: 40				• Average SMBG values
			C: 40				• Numbers of hypo- and hyperglycemic events.
Mauras [30]	USA	26 weeks	137	CGM (FreeStyle Navigator) vs. Usual care	Use CGM on a daily basis (Open) + ≥ 4 times SMBG	Use CGM on a daily basis (Blinded) + ≥ 4 times SMBG	• Decrease in HbA1c of ≥ 0.5% from baseline to 26 weeks with no severe hypoglycemia
			I: 69				
			C: 68				

Abbreviations: *AUC* area under the curve, *BG* blood glucose, *CGM* continuous glucose monitoring, *HbA1c* Hemoglobin A1c, *JDRF* Juvenile Diabetes Research Foundation, *MDI* multiple daily injection, *RT-CGM* real-time CGM, *SMBG* self monitoring of blood glucose.

**Figure 2:**
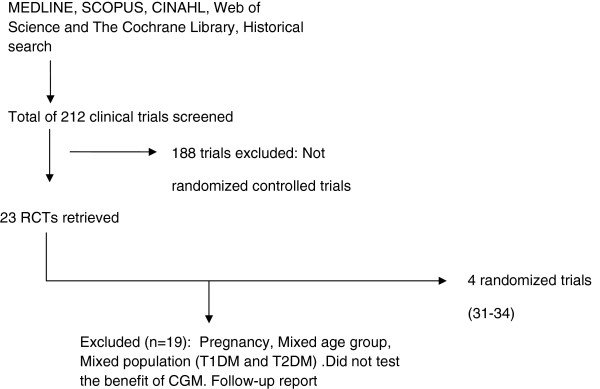
Summary of trial flow (Type 2 diabetic adults).

In Type 2 diabetes, five RCTs involving 161 participants met our predefined inclusion criteria [31-35]. The diagram of data extraction is illustrated in Figure 2. Among these 5 trials, there was a study which analyzed separately for two follow-up periods; i.e, 26 weeks and 52 weeks, and presented the results in two separated papers [34,35]. Only the paper with intervention period was included in the analysis [34]. Therefore, only 4 randomized trials were included in meta-analysis. The characteristics of included trials are summarized in Table 2. Two studies were of high quality [31,33] and other two studies were of low quality [32,34]. Among 4 trials, only one trial is multi-centered study [32]. One study was conducted to test the effects of a counseling intervention using continuous glucose monitoring system feedback on physical activity self-efficacy and reported HbA1c as a secondary outcome [33]. Two studies were performed in T2DM patients who were not treated with insulin [33,34] and other two included participants treated with either oral hypoglycemic agents (OHA) or insulin injections [31,32].

**Table 2:** Characteristics of included studies in Type 2 diabetic adults

**Study**	**Country**	**Duration**	**n**	**Intervention**	**CGM Use**	**SMBG Use**	**Outcomes**
Ehrhardt [34]	USA	3 months	100	RT-CGM (DexCom SEVEN) vs. SMBG	RT-CGM occurred in four cycles (2 weeks on/1 week off) for 3 months + SMBG before meals, at bedtime and at the time of hypo- or hyperglycemia	SMBG before meals and at bedtime, at the time of hypo- or hyperglycemia	• A1C
			I:50				• Change in mean and distribution of blood glucose
			C:50				
							• Weight
							• Blood Pressure
							• Diabetes – related stress
Cosson [32]	France	3 months	25	RT-CGM (The GlucoDay system) vs. SMBG	48 hour of CGM at baseline and after 3 months + usual SMBG	Usual SMBG	• A1C
							• Compare the 48 h CGM data at baseline with those obtained after 3 months:
			I:11				
			C:14				
							- Glucose control
							- Glucose variability
							- Hypoglycemia
Allen [33]	USA	8 weeks	46	RT-CGM vs. SMBG	72 hour of CGM + SMBG	SMBG	• Physical activity self efficacy
			I:21				
			C:25				• Physical activity levels
							• Blood pressure
							• Body mass index
							• A1C
Yoo [31]	Korea	3 months	57	RT-CGM(Guardian RT) vs. SMBG	Once a month for 3 day for 12 weeks + at least three SMBG per day	SMBG at least four times a week, including fasting blood glucose and postprandial 2 h blood glucose levels for 3 months continuously	• A1C
			I: 29				• Fasting blood glucose
			C:28				
							• Post prandial 2 h blood glucose
							• Lipid profiles
							• Weight
							• Waist circumference
							• Body mass index

Abbreviations: *RT-CGM* real-time CGM, *SMBG* self monitoring of blood glucose.

### Type 1 diabetic pediatrics

#### Total analysis

**Figure 3:**
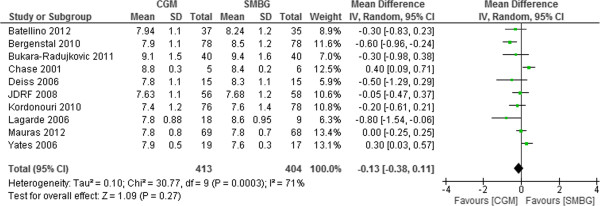
Mean difference (95% confidence interval) in HbA1c for CGM versus SMBG in T1DM pediatrics.

**Figure 4:**
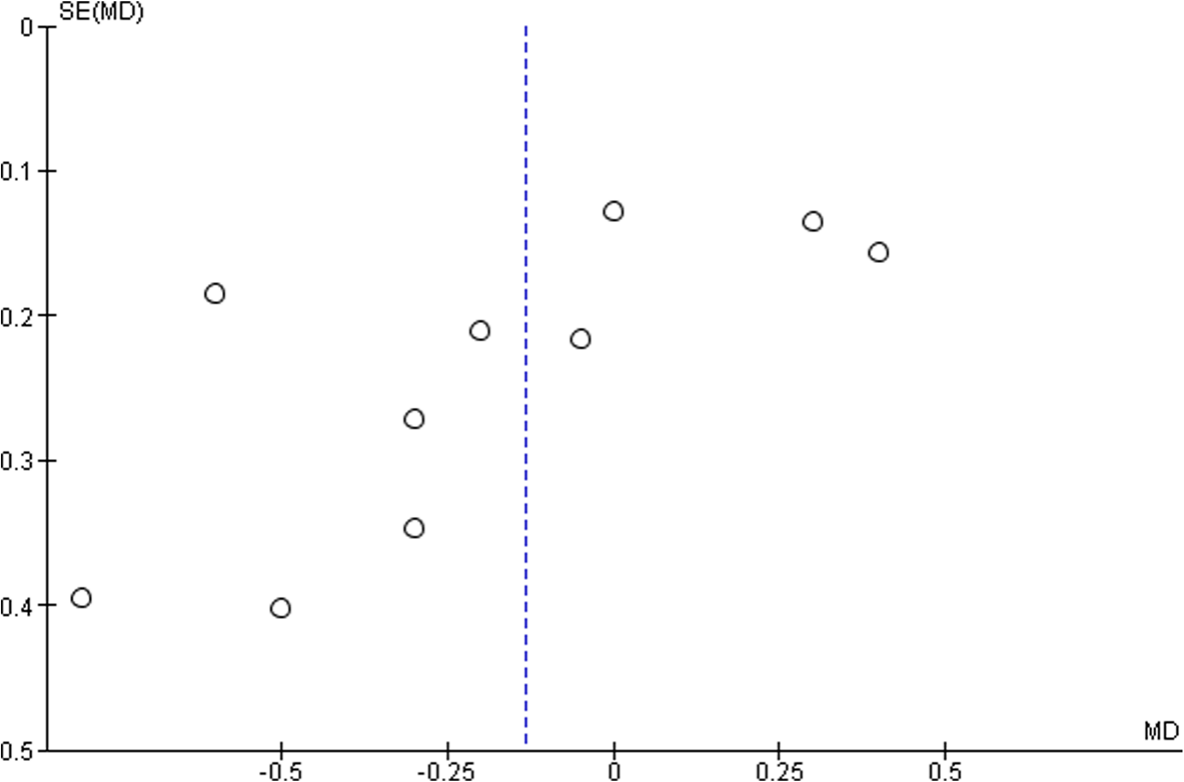
Funnel plot for ten randomized controlled trials of CGM versus SMBG in T1DM pediatrics.

There were a total of 817 type 1 diabetic pediatrics (413 in CGM group and 404 in SMBG group) in ten trials that compared CGM use with SMBG. Significant heterogeneity was found among the study results. The reason may be because of variations in patients’ characteristics, intervention used, pattern and frequency of CGM use, and quality of studies. The use of CGM did not provide better efficacy than SMBG use in terms of HbA1c reduction in T1DM pediatrics. The pooled estimate of HbA1c difference was – 0.13% (95%CI -0.38% to 0.11%, p = 0.27) (Figure 3). Publication bias was detected (Egger: bias = -3.19 (95% CI = -6.35 to -.028) P = 0.048 (Figure 4).

### Subgroup analysis based on type of data presentation

#### Retrospective CGM vs. SMBG

**Figure 5:**
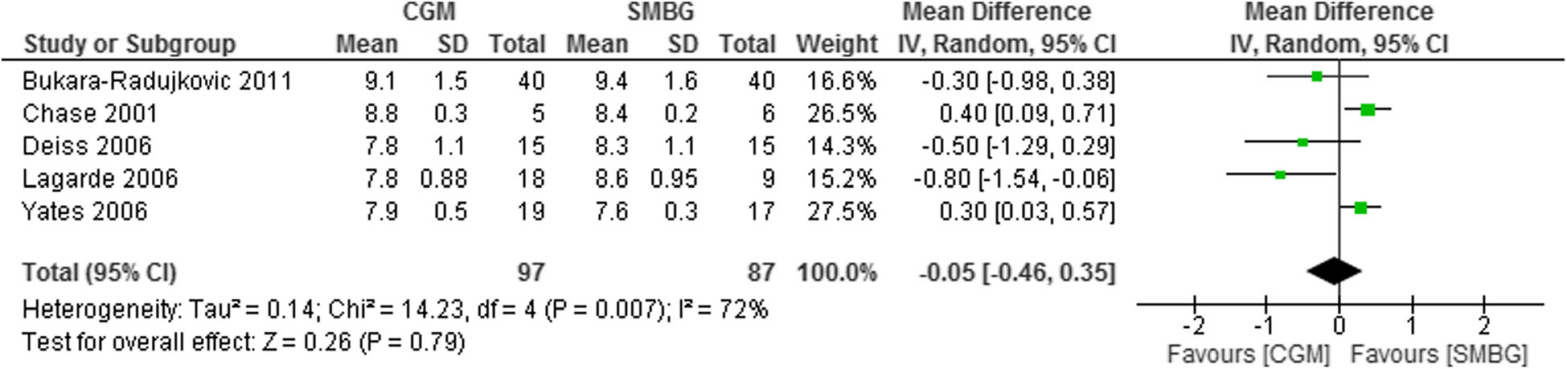
Mean difference (95% confidence interval) in HbA1c for retrospective CGM versus SMBG in T1DM pediatrics.

Retrospective CGM (r-CGM) was used in 5 trials [23-25,28,29]. The r-CGM was no better than SMBG use for glycemic control in T1DM pediatrics. The pooled mean difference in HbA1c was -0.05% (95% CI -0.46% to 0.35%, p = 0.79) (Figure 5).

#### Real-time CGM vs. SMBG

**Figure 6:**
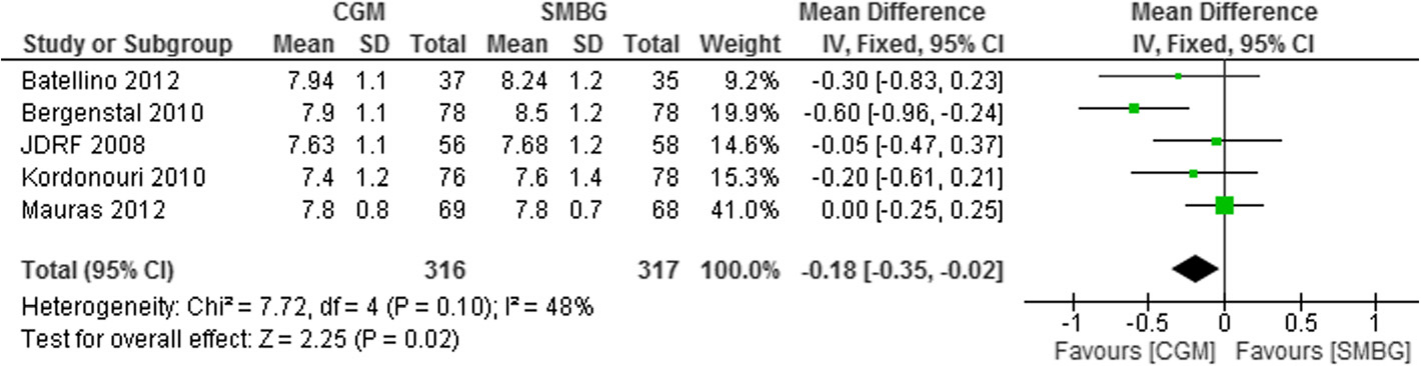
Mean difference (95% confidence interval) in HbA1c for real-time CGM versus SMBG in T1DM pediatrics.

Real time CGM (RT-CGM) was used in 5 trials [14,15,26,27,30]. The use of RT-CGM was superior to the usual care in improving glycemic control. The pooled mean difference in HbA1c was -0.18% (95% CI -0.35% to -0.02%, p = 0.02) (Figure 6).

### Subgroup analysis based on HbA1c at baseline

**Figure 7:**
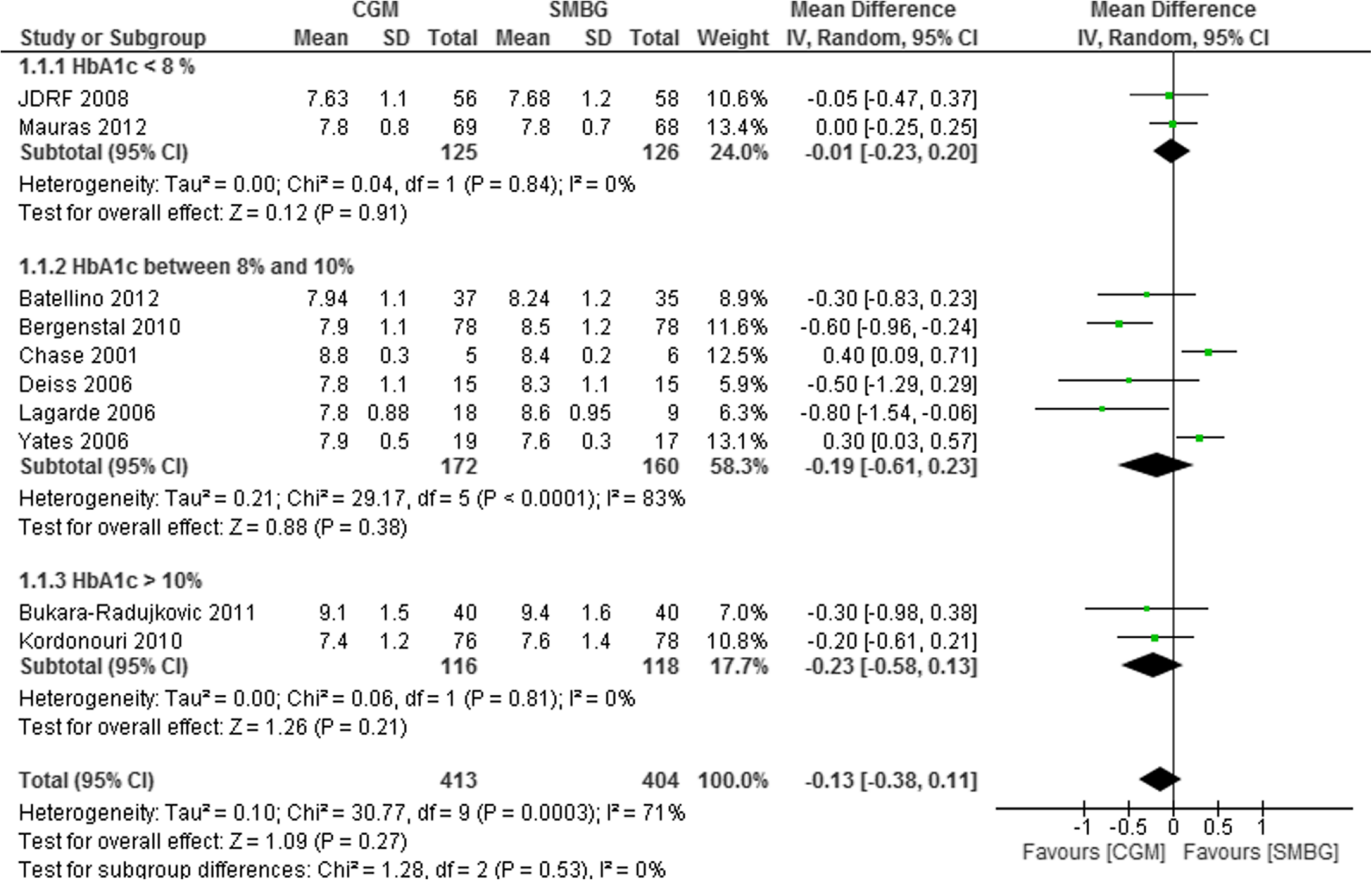
Mean difference (95% confidence interval) in HbA1c for CGM versus SMBG according to HbA1c at baseline in T1DM pediatrics.

Subgroup analysis was also performed according to baseline HbA1c of participants in the studies (HbA1c < 8%, HbA1c 8-10%, HbA1c > 10%). There were 2 studies with HbA1c at baseline < 8%. The results suggested that CGM was no more effective than SMBG in glycemic control regardless of baseline HbA1c level. The pooled mean difference in HbA1c was -0.01% (95%CI -0.23% to 0.20%, p = 0.91), -0.19% (95%CI -0.61% to 0.23%, p = 0.38) and -0.23% (95%CI -0.58% to 0.13%, p = 0.21), respectively, for baseline HbA1c < 8%, 8-10%, and > 10% (Figure 7).

### Subgroup analysis based on quality of included studies

**Figure 8:**
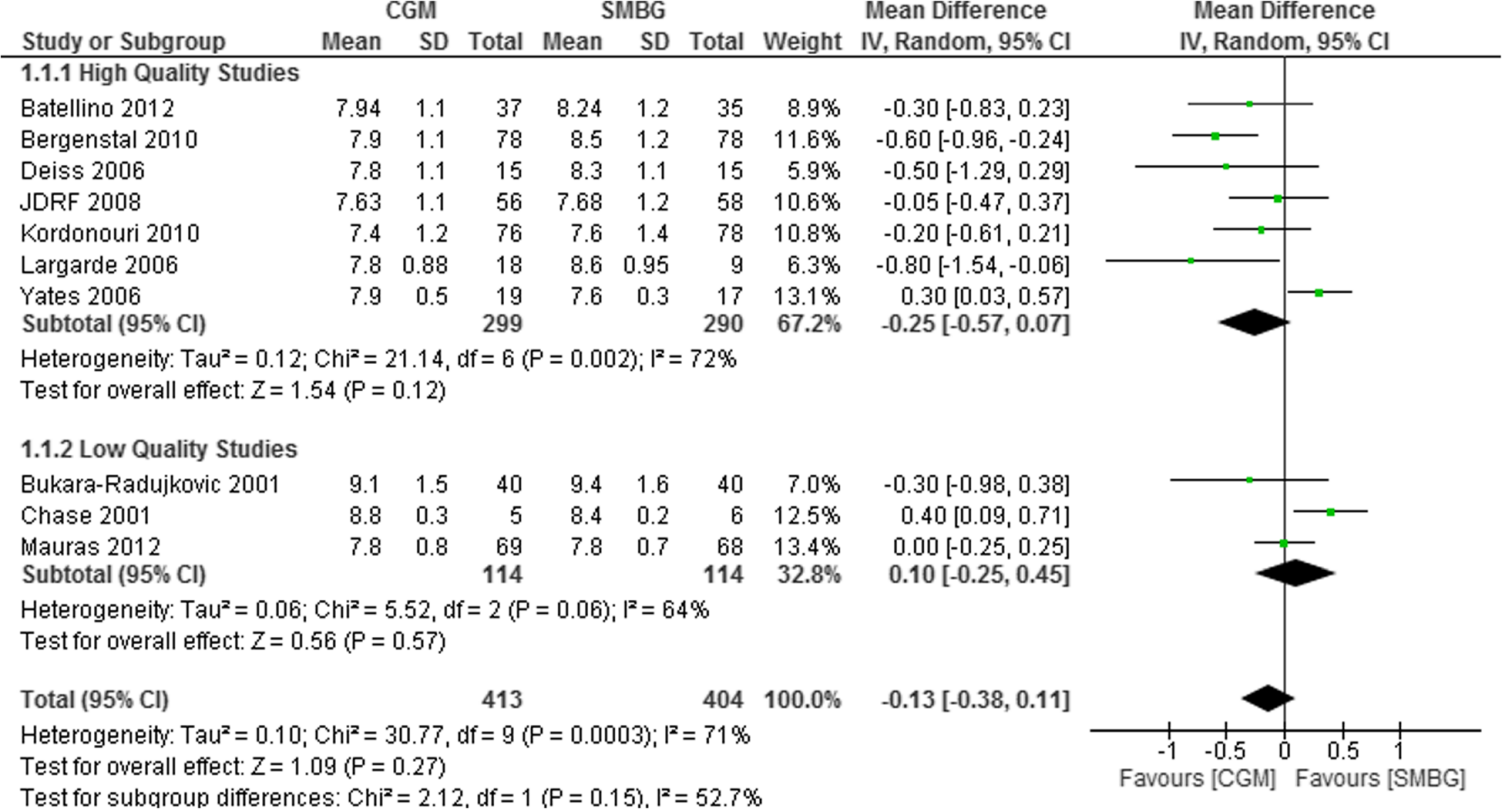
Mean difference (95% confidence interval) in HbA1c for CGM versus SMBG according to quality of included studies in T1DM pediatrics.

Subgroup analysis was also performed according to quality of studies (high quality studies and low quality studies). There were 7 high quality studies and 3 low quality studies. The pooled mean difference in HbA1c was -0.25% (95%CI -0.57% to 0.07%, p = 0.12) for high quality studies and 0.18% (95%CI -0.16% to 0.52%, p = 0.30) for studies with low quality (Figure 8).

### Sensitivity analysis

Sensitivity analysis was performed by excluding two studies which described lower number of usable CGM data [25,28]. For example, one study reported that only 70% of the total hours worn was usable and this suboptimal data was because of insufficient calibration points [25], while the other described that the lower numbers of usable CGM data was available because of patient noncompliance [28]. Participants in Chase et al. study [28] attained an average of less than 50% (421 of 864 possible) usable readings per sensor. We have doubted that whether this percentage of data would represent the overall effect on experimental population. Enough explanation for usable data was not provided in individual study and there are no criteria to identify the amount of the CGM data to represent the experimental group. Therefore, this may bias the result in favor of SMBG. Heterogeneity was reduced from 71% to 35% when these two trials were excluded. Glycemic control was better with CGM than SMBG (mean difference -0.23%, 95% CI -0.38% to -0.08%, p = 0.003) (Figure not shown).

### Type 2 diabetic adults

**Figure 9:**
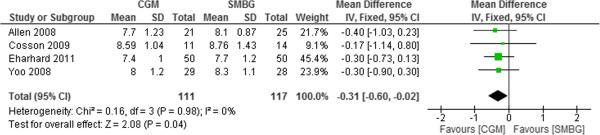
Mean difference (95% confidence interval) in HbA1c for CGM versus SMBG in T2DM adults.

There were a total of 228 type 2 diabetic adults (111 in the intervention group and 117 in the control group) in four trials that compared CGM use with SMBG. The use of CGM provided better efficacy than SMBG use in terms of HbA1c reduction in T2DM adults. The pooled mean difference in HbA1c was – 0.31% (95%CI -0.6% to -0.02%, p = 0.04) (Figure 9). No publication bias was detected (Egger: bias = 0.29, 95% CI = -1.77 to 2.36) (Figure not shown).

## Discussion

### Type 1 diabetic pediatrics

According to American Academy of Pediatrics, the term ‘pediatric’ includes population from birth to 18 years old [36]. In our meta-analysis, we then used the term ‘pediatric’ and included studies with T1DM participant ≤ 18 years of age. The principal meta-analysis evaluated the effect of both retrospective CGM and real-time CGM altogether and included 10 randomized controlled trials. The pooled results revealed that as a whole CGM was no more effective than SMBG in lowering HbA1c [mean difference -0.13%, 95%CI -0.38% to 0.11%). The results support the conclusion previously reported by Golicki et al (mean difference -0.02%, 95%CI -0.29 to 0.25) [16]. Their meta-analysis of CGM in children included five studies from the age of 2 to 19 years [16]. Our meta-analysis included ten studies, four of which already included in their meta-analysis. One study that was included in the previous meta-analysis was excluded from ours because participants were older than 18 years [37]. Although study participants aged < 18 years were eligible, those aged 2-18 years were enrolled among the trials contributed to our meta-analysis since there was no randomized study on participants with age ≤ 2 years. In addition, their meta-analysis limited to only one device, Metronic MiniMed. In contrast, our meta-analysis included all kinds of CGM devices which are available in the markets in the USA or Europe since the purpose of our study was to investigate a treatment strategy using CGM technology and not a specific device.

Inconsistency among studies as assessed by I^2^ statistic was statistically significant (I^2^ = 71%). There was difference in sample size among studies ranging from 11 to 156 participants. Other possible reasons for heterogeneity may be the differences in frequency and duration of CGM use, intervention period, and intervention used among those studies (retrospective or real-time CGM studies). In all studies with retrospective CGM, the device was used for 72 consecutive hours but with different frequency for different duration, for example, 6 times in 1 month [28], 1 time in 3 months [29], 4 times in 3 months [25], 3 times in 4 months [24], and 1 time in 3 months [23]. For RT-CGM, the device was used on a daily basis except in the study by Bergenstal et al. [14] in which the device was used for 1-week period continuously per 6 months. Subgroup analysis based on the frequency of CGM use was not possible. In addition, various types of insulin delivery systems were employed among individual trials, for example, both insulin pump and insulin injection therapies [24-26,28,30], insulin injection alone [14,23,29], and insulin pump therapy alone [15,17].

It has been demonstrated that patients who used CGM devices had a greater decrease in hemoglobin A1c from baseline compared with those using SMBG in type 1 diabetics patients [mean difference – 0.26% (95%CI -0.34% to -0.19%)]. However, when the effectiveness of the r-CGM and RT-CGM compared with SMBG were analyzed separately, only RT-devices for CGM improved glycemic control [mean difference -0.27% (95%CI-0.34% to -0.19%)] [38]. The beneficial effect of RT- CGM over SMBG was consistently described in type 1 pediatric population [39]. Other meta-analysis of CGM in pediatric population also reported no significant effect of r- CGM compared with SMBG [16]. Therefore, although RT-CGM gave profound benefit to type 1 diabetic population, the value of r-CGM was somewhat limited. These results are subsequently confirmed by our meta-analysis, showing that as a whole CGM did not provide better benefit over SMBG. Specifically, glycemic control was better with RT-time CGM compared with SMBG [mean difference -0.18% (95% CI -0.35% to -0.02%, p = 0.02)], whereas it was no better with r-CGM than with SMBG [mean difference -0.05% (95% CI -0.46% to 0.35%)]. As a matter of fact, regarding patient perspective, learning about the application of device can be a burden to patients in using RT-CGM. R-CGM seems to be more convenient since patients need doing nothing. However, RT-CGM proved better glycemic control than r-CGM. For r-CGM, physicians or care providers can adjust therapy based on CGM data. For RT-CGM, physicians make therapeutic adjustment based on CGM data and, at the same time, patients can also monitor lifestyle intervention themselves. In other words, RT-CGM can be a supportive tool for both pharmacological intervention and lifestyle intervention. This contributes to better glycemic control compared with r-CGM.

One important factor in judging the efficacy of glucose-lowering therapies is baseline HbA1c, as higher baseline HbA1c is associated with greater reductions in HbA1c [40] if tightly controlled. Regarding the effect of SMBG, glycemic control significantly improved in patients whose baseline HbA1c was ≥ 8% [9]. The impact of baseline HbA1c on the effect of CGM in lowering glucose level has never been evaluated yet. In our meta-analysis, participants from the included studies had varied baseline HbA1c level. Diabetes Center and Joslin Clinic made a clinical recommendation that intensive therapy is need if HbA1c is > 8% [41]. Moreover, significant high risk of microvascular complications was found in subjects with poor glycemic control (HbA1c > 8%) [42]. Therefore, we used HbA1c level of 8% as a cutoff point and the analysis was stratified into three subgroups of baseline HbA1c: <8%, 8 - 10% and > 10%. It was found that CGM was not effective in improving glycemic control regardless of baseline HbA1c. Moreover, subgroup analysis by quality of studies was performed to analyze the effect of study quality on heterogeneity and it was found that quality of studies had no impact on heterogeneity.

The Juvenile Diabetes Research Foundation primary study which compared RT- CGM with SMBG in three age groups (8-14 years, 15 – 24 years and ≥ 25 years) found that CGM was only significantly more effective than SMBG in those aged 25 or more, most likely because older patients used the sensor more frequently since it was found that sensor use was significantly greater in ≥ 25 age group than ≤ 25 age groups (p < 0.001) [26]. The important point related with this finding may be independent of sensor use; older patients are slightly more able than younger patients to interpret data from continuous glucose monitoring to adjust therapy and lifestyle intervention to maintain good glycemic control. To attain better glycemic control in pediatric population, co-operation of parents is demanded. Poor adherence in diabetes management has been recognized as a hindrance to get successful glycemic control in adolescents and children with type 1 diabetes. Moreover, the transition from parental assistance with management of diabetes to patient-only management is often associated with deterioration of glycemic control [43,44]. According to those findings, RT- CGM can be a useful tool in glycemic control for T1DM pediatric population if the parents and children are carefully educated about the application and benefit of the device. Among 5 RT-CGM studies included in our meta-analysis, only one study indicated that both patients and their parents were educated about managing the device [30], whereas only patients were educated in the remaining four studies. Better results from RT-CGM devices can be expected when not only patients but also their parents are carefully educated about the effect of device on therapy.

Apart from substantial heterogeneity among study results as previously discussed, the limitation of meta-analysis in T1DM pediatrics was the presence of publication bias since only published trials were included. This may lead to omitting appropriate data for the analysis. Other than publication bias, plot asymmetry may be due to small study effect; i.e, the association between estimated intervention effect and the sample size is much different from those might be expected by chance. Consequently, false positive results can be brought about by small studies. In our meta-analysis, we suspected small study effect to be contributed by one study in which the sample size was too small (n = 11) [28]. The effective appropriate frequency and duration of CGM use is also a good point to focus in further studies. In addition, it would be worth analyzing the benefit of CGM device in the populations with nocturnal hypoglycemia, hypoglycemic unawareness where the usefulness of SMBG is limited.

### Type 2 Diabetic Adults

It was found that CGM can be a useful tool to reduce HbA1c level in type 2 diabetes adults. Although no heterogeneity was detected (I^2^ = 0%), there were some variations among the included studies in terms of study quality, types of data presentation (retrospective and real-time), frequency and duration of CGM use, intervention used by the participants. Among four studies included, two studies used retrospective CGM [32,33] whereas other two studies used real-time CGM [31,34]. The frequency of CGM use differed greatly from trial to trial, making it difficult to suggest which one is the most appropriate frequency of CGM use. Generally, factors to be considered in determining the frequency of use will include the conditions of patients, i.e. whether their glycemia are poorly controlled or well controlled, and to what extent they are vulnerable to hypoglycemia. Participants were treated with either oral hypoglycemic agents only [33], prandial insulin [34] or both insulin and oral hypoglycemic agents [31,32].

According to 2006 American Diabetes Association’s recommendation, SMBG is regarded as an essential aspect of diabetes management in insulin-treated patients and a desirable aspect in non-insulin treated patients with diabetes [45]. Although, the effectiveness of SMBG in T2DM patients who do not use insulin had been provided in some meta-analyses, SMBG is not recommended yet. High risk of hypoglycemia can be predisposed by the use of insulin, whereas oral agents particularly metformin, thiazolidinediones, and DPP-4 inhibitors, are not prone to result in hypoglycemia. Moreover, SMBG data or CGM data are generally used to adjust insulin dose. Therefore, in non-insulin treated T2DM, the value of SMBG and CGM previously seems to be limited. In our review, participants in the study by Ehrhardt et al. [34] did not use prandial insulin and participants in the study by Allen et al. [33] did not use any type of insulin at all. These two studies reported the significant reduction in HbA1c in favor of CGM group. This may be because CGM data can provide benefit to lifestyle intervention other than adjusting insulin therapy. In the study by Allen et al. [33], CGM device was used as a counseling tool in evaluating the effect of physical activity on glycemic control and in the study by Ehrhardt et al. [34], patients were allowed to manage their lifestyle based on CGM data. In agreement with the result, CGM use in non-insulin treated T2DM may be beneficial. It is noted that patients enrolled in individual studies had baseline HbA1c > 8%. Thus, CGM devices may be a useful tool in poorly controlled T2DM patients.

When the effectiveness of the r-CGM and RT-CGM compared with SMBG were analyzed separately in T2DM patients, only RT-CGM devices improved glycemic control [mean difference -0.27% (95%CI-0.34 to -0.19)]. One systematic review concluded that RT-CGM may be more effective than r-CGM in type 1 diabetes [39]. However, in type 2 diabetes, whether retrospective CGM or real-time CGM provides superior benefit in lowering HbA1c level compared with SMBG is not known yet. In our meta-analysis, two studies used RT-CGM (Yoo et al. and Ehrhardt et al.) [31,34] and two studies analyzed data in a retrospective manner (Allen et al. and Cosson et al.) [32,33]. Both RT-CGM studies and one r-CGM study provided significant difference in lowering HbA1c level by CGM compared with SMBG. However, there is a confounding factor in the study used r-CGM. This r-CGM study was performed to test the role of CGM feedback in physical activity counseling. Therefore, all participants in the study were in physical activity program and these participants were educated about the benefit of physical activity using individual CGM graph or profile at the end of study. As a result, it is difficult to say that lowering HbA1c level was due to CGM device since there was an impact of physical activity on HbA1c level. Therefore, it may be possible that positive effect in our meta-analysis was contributed merely by RT-CGM. Unfortunately, subgroup analysis for quantitative examination of the effect of r-CGM and RT-CGM cannot be performed since the number of studies was too small.

In addition to pharmacological intervention, lifestyle intervention is already accepted as an important aspect in the management of type 2 diabetes. Patients with lifestyle intervention experience significant improvement in blood glucose and lipid profile after one year compared with those without this intervention, supporting the value of lifestyle intervention in T2DM management [46]. Continuous glucose monitoring can provide glucose information promptly to adjust the dosages of medication, and/or to manage the dietary or exercise regimen, which could lead to better lifestyle for diabetes patients. In other words, the glycemic values from CGM devices allow care provider or patient to encounter the noticeable effects of meals and exercise on glycemic level and to manage lifestyle skills that results in better glycemic control. Moreover, from the glycemic values displayed by CGM devices, patients can be easily educated about the effects of their medication and lifestyle on diabetes. A study testing the effects of counseling intervention of physical activity and using continuous glucose monitoring system feedback suggested that counseling T2DM patients with CGM feedback may improve physical activity level and, as a result, diabetes-related complications could be reduced [33]. Therefore, with the help of continuous glucose monitoring, not only pharmacological intervention can be adjusted, but non-pharmacological intervention can also be promoted.

There were some limitations in the meta-analysis of type 2 diabetes. We did not search for unpublished trials. Thus there was a possibility of missing relevant data. However, no publication bias was detected. The average baseline HbA1c of participants from all studies was above 8%. Therefore, the routine use of CGM in patients with uncontrolled T2DM may provide benefit and deserves consideration. More randomized trials with adequate sample sized are needed for CGM use in type 2 diabetes population. In addition, further studies are suggested to focus on the frequency and duration of RT-CGM use to achieve the legacy effects.

## Conclusion

### Type 1 diabetic pediatrics

The available evidence from this meta-analysis suggests that CGM use was no more effective than SMBG in reducing HbA1c in type 1 diabetic pediatrics. However, the results of subgroup analyses showed that RT-CGM can be more effective than SMBG in reducing HbA1c. In contrast, r-CGM was no better than SMBG in T1DM pediatric population. Therefore, RT-CGM devices can be an effective tool in Type 1 diabetic pediatrics population.

### Type 2 diabetic adults

Our results suggest that the effect of CGM use in lowering HbA1c level was superior to SMBG for type 2 diabetes adults. Since the average baseline HbA1c of participants from all studies was above 8%, CGM device can be an effective tool in patients with uncontrolled T2DM. However, the comprehensive randomized controlled trial need to be established in order to address the long term benefit of CGM.

## Abbreviations

ADA: American diabetes association; AUC: Area under the curve; BG: Blood glucose; CGM: Continuous glucose monitoring; HbA1c: Hemoglobin A1c; IDDM: Insulin dependent diabetes mellitus; NIDDM: Non-insulin dependent diabetes mellitus; OHA: Oral hypoglycemic agents; RCT: Randomized controlled trial; r-CGM: retrospective CGM; RT-CGM: Real-time CGM; SMBG: Self monitoring of blood glucose; T1DM: Type 1 Diabetes Mellitus; T2DM: Type 2 Diabetes mellitus; UKPDS: UK Prospective diabetes study; 95% CI: 95% Confidence interval.

## Competing interests

The authors declare that they have no competing interests.

## Authors’ contribution

NS contributed to conception, design, analysis and interpretation of data and drafted the manuscript. NP and AMK contributed to design, analysis and interpretation of data and drafted the manuscript. All authors read and approved the final manuscript.
